# Variation in Survival After Dementia Diagnosis by Sex, Race/Ethnicity, and Geography

**DOI:** 10.1002/alz.090914

**Published:** 2025-01-09

**Authors:** Maria J. Lopez, Haiqun Lin, Anum Zafar, Hyosin Kim, Weiyi Xia, Olga F Jarrín Montaner

**Affiliations:** ^1^ Rutgers, The State University of New Jersey, New Brunswick, NJ USA; ^2^ Rutgers, The State University of New Jersey, Newark, NJ USA; ^3^ Oregon State University, Corvallis, OR USA; ^4^ Rutgers, The State University of New Jersey, Piscataway, NJ USA

## Abstract

**Background:**

Dramatically varying estimates of survival time following an initial diagnosis of Alzheimer’s disease and other dementias have been reported across different studies. The prevalence of dementia increases with age while survival time decreases with increasing age at diagnosis. This study aimed to investigate how survival time after a diagnosis of dementia varies by sex, race/ethnicity, and age at time of diagnosis.

**Method:**

A retrospective analysis of the Centers for Medicare & Medicaid Services Chronic Conditions Warehouse 100% Master Beneficiary Summary File was conducted for Medicare beneficiaries aged 50 and older who died in 2019 and had a dementia diagnosis date (n = 884,548). Survival time in years and associations with sex, race/ethnicity, age at diagnosis, clinical and sociodemographic factors, and state of residence were investigated. Descriptive statistics, generalized gamma regression survival‐time models, and Kitagawa‐Blinder‐Oaxaca decomposition of racial/ethnic differences were conducted.

**Result:**

Among the 2019 Medicare decedents with dementia, the median survival time in years was shorter for American Indian/Alaska Native (AIAN) (2.7, IQR 0.8‐6.0) and White (2.9, IQR 0.8‐6.3) beneficiaries, and longer for Black (3.4, IQR 0.9‐7.4), Asian American/Pacific Islander (AAPI) (3.6, IQR 1.1‐7.8), and Hispanic (3.8, IQR 1.1‐8.5) older adults. Median survival time also varied by sex and age at dementia diagnosis. Hispanic and AAPI females diagnosed with dementia before age 80 had a median survival time exceeding 6 years after a dementia diagnosis. Median survival time also varied by geographical region and was notably longest for female Hispanic decedents in Puerto Rico (9.0, IQR 2.5‐14.7). Using the Kitagawa‐Blinder‐Oaxaca modeling decomposition approach, clinical and sociodemographic predictors fully explained the difference in survival time between White, Black, and AIAN individuals; however, more than half of the difference in longer survival time for Hispanic and AAPI individuals was unexplained.

**Conclusion:**

Hispanic and AAPI older adults live significantly longer after receiving a diagnosis of dementia compared to White, Black, and AIAN older adults. Unexplained differences may be associated with cultural factors, family and social support systems, healthcare access and utilization, environmental factors, and differences in diagnosis rates and timing of diagnosis.

